# *Blautia*—a new functional genus with potential probiotic properties?

**DOI:** 10.1080/19490976.2021.1875796

**Published:** 2021-02-01

**Authors:** Xuemei Liu, Bingyong Mao, Jiayu Gu, Jiaying Wu, Shumao Cui, Gang Wang, Jianxin Zhao, Hao Zhang, Wei Chen

**Affiliations:** a State Key Laboratory of Food Science and Technology, Jiangnan University, Wuxi, Jiangsu, China; b School of Food Science and Technology, Jiangnan University, Wuxi, Jiangsu, China; c National Engineering Research Center for Functional Food, Jiangnan University, Wuxi, Jiangsu, China; d Beijing Innovation Center of Food Nutrition and Human Health, Beijing Technology and Business University (BTBU), Beijing, China

**Keywords:** *Blautia*, biotransformation, probiotics, host health

## Abstract

*Blautia* is a genus of anaerobic bacteria with probiotic characteristics that occur widely in the feces and intestines of mammals. Based on phenotypic and phylogenetic analyses, some species in the genera *Clostridium* and *Ruminococcus* have been reclassified as *Blautia*, so to date, there are 20 new species with valid published names in this genus. An extensive body of research has recently focused on the probiotic effects of this genus, such as biological transformation and its ability to regulate host health and alleviate metabolic syndrome. This article reviews the origin and biological characteristics of *Blautia* and the factors that affect its abundance and discusses its role in host health, thus laying a theoretical foundation for the development of new functional microorganisms with probiotic properties.

## Introduction

The gastrointestinal tract of mammals harbors a complex and diverse community of microorganisms, including a variety of bacteria, viruses, archaea, and eukaryotes.^1^ According to the results of previous 16S rRNA studies, Firmicutes is one of the core phyla of the intestinal microbiota of humans and other vertebrates, and *Lachnospiraceae* and *Ruminococcaceae* are the most abundant families of this phylum, accounting for 50% and 30% of the total intestinal microbiota, respectively.^2^ As a genus of the *Lachnospiraceae* family, *Blautia* has been of particular interest since its establishment for its contribution to alleviating inflammatory diseases and metabolic diseases and for its antibacterial activity against specific microorganisms.^3,4^ Several recent reports have indicated that the composition of and changes in the *Blautia* population in the intestine are related to factors such as host age, geography, diet, genotype, health, disease state, and other physiological states.^5–7^ This genus has also been revealed to play a certain role in biotransformation and crosstalk with other intestinal microorganisms.^8,9^ Although *Blautia* has shown a series of potential probiotic properties, there is no comprehensive understanding of this genus, probably due to the lack of a comprehensive review. The Ninth Edition of Bergey’s Manual of Systematic Bacteriology contains no description of the genus *Blautia*, only some introduction to the previous species belonging to the genus *Clostridium* or *Ruminococcus* and a summary of the characteristics of the type strains. With the development and popularization of high-throughput sequencing technology, increasing numbers of new species from the gut and feces are being isolated and identified, so a more comprehensive description of this new genus is urgently warranted. This article reviews the recent studies on *Blautia* and discusses its potential probiotic role in host health.

## Morphological, physiological, and biochemical characteristics of *Blautia*

**Figure 1.**
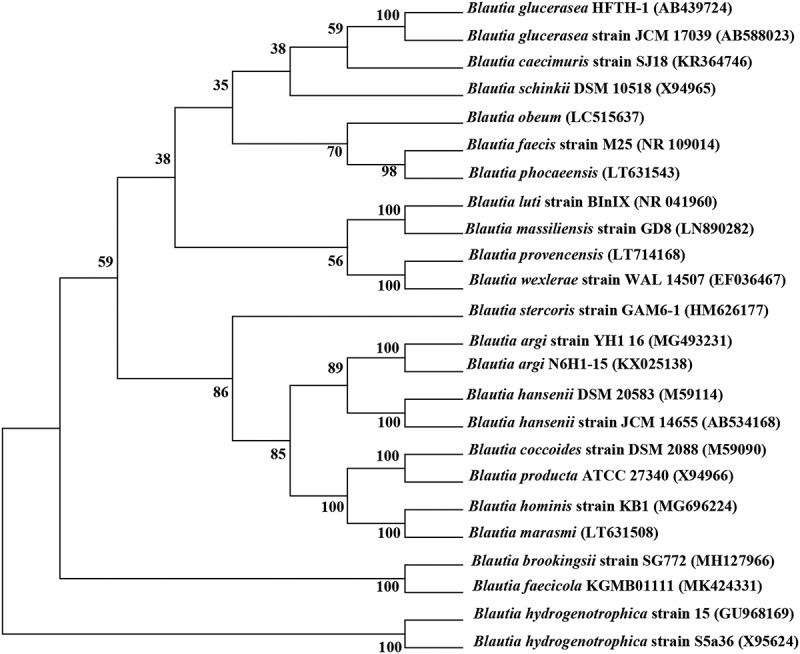
Phylogenetic consensus tree based on 16S rRNA gene sequences, reconstructed with the neighbor-joining (NJ), maximum-parsimony (MP) and maximum-likelihood (ML) algorithms. Bootstrap values calculated for 1000 subsets are shown at branch nodes

As opposed to traditional culture methods alone, phylogenetic analysis of 16S rRNA gene sequences in combination with culture-based analyses is an effective approach to identify microbial diversity in the host intestine and feces.^10^ Using phenotypic and phylogenetic analyses, Liu et al^11^ revealed a hitherto unknown coccus-shaped strain WAL 14507 T (=ATCC BAA-1564 T = DSM 19850 T) and established it as the new species *Blautia wexlerae* sp. nov. To date, 20 species with valid published names constitute the genus *Blautia* (Table 1), including *B. coccoides, B. hansenii*, and *B. producta*, which were originally misclassified into the genus *Clostridium* or *Ruminococcus*.^11^ A phylogenetic tree based on the 16S rRNA gene sequences of the representative species of *Blautia* is provided in Figure 1. The composition of this genus is constantly updated by adding new species and strains, but in general, the species in *Blautia* still form a relatively stable and coherent uniline branch.^27^

**Table 1.** All species of *Blautia* reported in the literature

Strains	Isolation source	Gram Staining	Type strain	References
*Blautia coccoides* (*Clostridium coccoides*)	mice feces	G^+^	ATCC 29236 T	Kaneuchi et al.(1976)^12^
*Blautia hansenii* (*Ruminococcus hansenii*)	human feces	G^+^	ATCC 27752 T	Holdeman et al.(1974)^13^
*Blautia hydrogenotrophica* (*Ruminococcus hydrogenotrophicus*)	human feces	G^+^	DSM 10507 T	Bernalier et al.(1996)^14^
*Blautia luti* (*Ruminococcus luti*)	human feces	G^+^	DSM 14534 T	Simmering et al.(2002)^15^
*Blautia producta* (*Ruminococcus productus*)	sputum	G^+^	ATCC 27340 T	Ezaki et al.(1994)^16^
*Blautia schinkii* (*Ruminococcus schinkii*)	rumen of lambs	G^+^	DSMZ 105/8	Rieu-Lesme et al. (1996)^17^
*Blautia wexlerae*	children’s stool	G^+^	ATCC BAA-1564 T	Liu et al.(2008)^11^
*Blautia glucerasea*	dog feces	G^+^	DSM 22028 T	Furuya et al.(2010)^18^
*Blautia stercoris*	human feces	G^+^	KCTC 5981 T	Park et al.(2012)^19^
*Blautia faecis*	human feces	G^+^	KCTC 5980 T	Park et al.(2013)^20^
*Blautia obeum* (*Ruminococcus obeum*)	human feces	G^+^	ATCC 29174 T	Moore et al.(1976)^21^
*Blautia caecimuris*	mouse cecum	G^+^	SJ18T	Lagkouvardos et al.(2016)^22^
*Blautia massiliensis*	human fecces	G^−^	DSM 101187 T	Durand et al.(2017)^23^
*Blautia phocaeensis*	human feces	G^+^	Marseille-P3441	Traore et al.(2017)^24^
*Blautia marasmi*	Human stool	G^+^	Marseille-P2377T	Pham et al.(2017)^25^
*Blautia provencensis*	children’s stool	G^+^	Marseille-P3502T	Pham et al.(2017)^26^
*Blautia hominis*	human feces	G^+^	KCTC 15618 T	Shin et al.(2018)^27^
*Blautia argi*	dog feces	G^+^	KCTC 15426 T	Paek et al.(2019)^28^
*Blautia brookingsii*	human feces	G^+^	SG772	Ghimire et al.(2020)^29^
*Blautia faecicola*	human feces	G^+^	KGMB01111 T	Kim et al.(2020)^30^

According to reports, the human intestinal microbial community can be divided into three “enterotypes,” namely, *Bacteroides, Prevotella*, and *Ruminococcu*s, among which the *Ruminococcu*s type is mostly driven by related groups of the order Clostridiales, *Blautia*, and unclassified Lachnospiraceae.^31^ *Blautia* is widely distributed in mammalian feces and intestines. For example, *B. hydrogenotrophica* and *B. stercoris* were first isolated from human feces;^14,19^ *B. wexlerae* and *B. luti* were found to be the most abundant of the *Blautia* spp. and are considered among the dominant species of the human intestine;^32^ *B. coccoides* was first isolated from the feces of mice fed a high-lactose diet;^12^ *B. glucerasei* was isolated from the feces of dogs;^18^ and some species such as *B. producta* and *B. schinkii* were even isolated from sewage and rumen.^17,33^ These findings indicate the importance of the survival and evolution of *Blautia* in the gut and other microenvironments.

*Blautia* species are strictly anaerobic, non-motile, 1.0–1.5 × 1.0–3.0 μm in size, usually spherical or oval, and appear in pairs or strands, with most strains being sporeless. The optimum temperature and pH for most *Blautia* strains are 37°C and 7.0, respectively.^11^ Some species such as *B. producta* possess both heterotrophic and autotrophic properties and can use CO, H_2_/CO_2_, and carbohydrates as energy sources.^34^ Carbohydrate utilization experiments have shown that all *Blautia* strains can use glucose, but different strains showed different abilities to use sucrose, fructose, lactose, maltose, rhamnose, and raffinose (Table 2). The final products of glucose fermentation by *Blautia* are acetic acid, succinic acid, lactic acid, and ethanol, and the main biochemical tests have revealed negative results for lecithin, lipase, catalase, and indole. The long-chain fatty acids produced by *Blautia* strains are classified into linearly saturated and monounsaturated types, with C14:0, C16:0, and C16:00 dimethyl acetal fatty acids as the main species. The GC content of *Blautia* DNA is 37–47 mol%, and the type species of this genus is *B. coccoides*.^11^

**Table 2.** Characteristics of carbohydrates utilization in *Blautia* strain

Strains	*Blautia coccoides* CLC-1	*Blautia hansenii* ATCC 27752 T	*Blautia hydrogenotrophica* S5a33	*Blautia luti* BInIXT	*Blautia producta* U-1	*Blautia schinkii* strain B	*Blautia wexlerae* WAL 14507 T	*Blautia glucerasei* HFTH-1 T	*Blautia stercoris* GAM6-1 T	*Blautia faecis* M25T	*Blautia obeum* ATCC 29174 T	*Blautia caecimuris*	*Blautia massiliensis* GD9T	*Blautia hominis* KB1T	*Blautia argi* N6H1-15 T	*Blautia brookingsii* SG-772	*Blautia faecicola.*
End products of fermentation	S,A	L,A	A,L	A,S	A,L,	A,S	A,S	A,F	A	L,A	A	ND	ND	A,S	A,L	ND	A
Glucose	+	+	+	+	+	+	+	+	+	+	+	+	+	+	+	+	-
Mannose	+	-	w	+	ND	+	+	-	-	-	+	ND	+	+	-	+	-
Arabinose	+	-	-	+	+	+	+	+	+	+	+	+	+	+	+	ND	w
Xylose	+	-	ND	+	+	+	+	+	-	-	+	+	+	+	-	ND	-
Robise	+	-	ND	+	+	+	+	ND	+	+	+	ND	ND	+	-	ND	-
Sucrose	+	-	-	+	+	+	D	ND	-	-	-	+	+	+	+	ND	w
Fructose	+	-	+	+	+	+	D	+	-	-	+	ND	ND	+	+	ND	-
Lactose	+	+	ND	+	+	ND	D	+	-	-	+	+	+	+	+	ND	-
Maltose	+	+		+	+	+	D	+	-	-	+	+	+	+	+	ND	-
Cellobiose	+	-	+	+	+	+	D	+	-	-	+	ND	+	+	+	+	-
Rhamnose	+	-	-	-	+	ND	D	ND	-	-	+	+	+	+	-	+	-
Raffinose	+	+	ND	+	+	+	D	+	-	-	+	+	+	+	-	ND	w
Melezitose	ND	-	ND	-	+	ND	D	ND	-	-	ND	ND	+	+	-	+	w
Mannitol	+	-	-	-	+	ND	D	ND	-	-	-	ND	+	-	-	+	-
Melibiose	+	w	-	+	+	+	D	ND	-	-	+	ND	ND	+	+	ND	-

+, Positive; –, negative; W, weakly positive; D,different among strains; ND, no data.

A, acetic acid; S, succinic acid; L,lactate.

Data are from Kaneuchi et al.(1976), Holdeman et al.(1974), Bernalier et al.(1996), Simmering et al.(2002), Lorowitz et al.(1984), Rieu-Lesme et al.(1996), Liu et al.(2008), Furuya et al. (2010), Park et al. (2012), Park et al. (2013), Moore et al. (1976), Lagkouvardos et al. (2016), Durand et al. (2017), Shin et al. (2018), Paek et al. (2019), Ghimire et al. (2020), Kim et al. (2020).

## Genomic and comparative genomic analysis of the members of *Blautia*

The high performance and efficiency of next-generation sequencing technology have allowed novel insights into the whole genome of several bacteria.^35^ Bioinformatics is widely used to analyze bacterial genome information, enabling the research direction shift from phenotypic assessments to genomic evaluations and even prediction of potential probiotic functions.^36^ Compared with the abundance of well-known probiotics such as *Bifidobacterium* and *Lactobacillus* species and of their genomic data, fewer *Blautia* species have been isolated, so information on their genome is limited. Currently, there are 12 isolated *Blautia* species with a total of 195 genome assemblies according to the NCBI database. The genome size varies greatly and ranges from 3.17 to 6.07 Mb with a median of 3.49 Mb. The median GC content is 44.25%, and the median protein count is 3205 (These data were obtained from the NCBI database on September 1, 2020, and detailed information is presented in Table 3).

**Table 3.** The genomic characteristics of *Blautia.*

Species	Number of genome assembly	Median total length (Mb)	Median GC (%)	Median protein count
*Blautia wexlerae*	81	4.07	41.20	3572
*Blautia obeum*	50	3.66	41.60	3302
*Blautia luti*	5	3.96	43.80	3469
*Blautia hansenii*	4	3.17	41.10	2916
*Blautia schinkii*	8	3.67	43.20	3165
*Blautia hydrogenotrophica*	3	3.53	44.90	3225
*Blautia producta*	12	6.07	45.70	5300
*Blautia marasmi*	2	6.02	46.30	5055
*Blautia coccoides*	3	5.97	45.60	5156
*Blautia faecis*	6	4.53	42.80	3929
*Blautia glucerasea*	3	4.31	43.00	3738
*Blautia massiliensis*	18	3.49	44.10	3072

Data were obtained from NCBI database on September 1, 2020.

**Figure 2.**
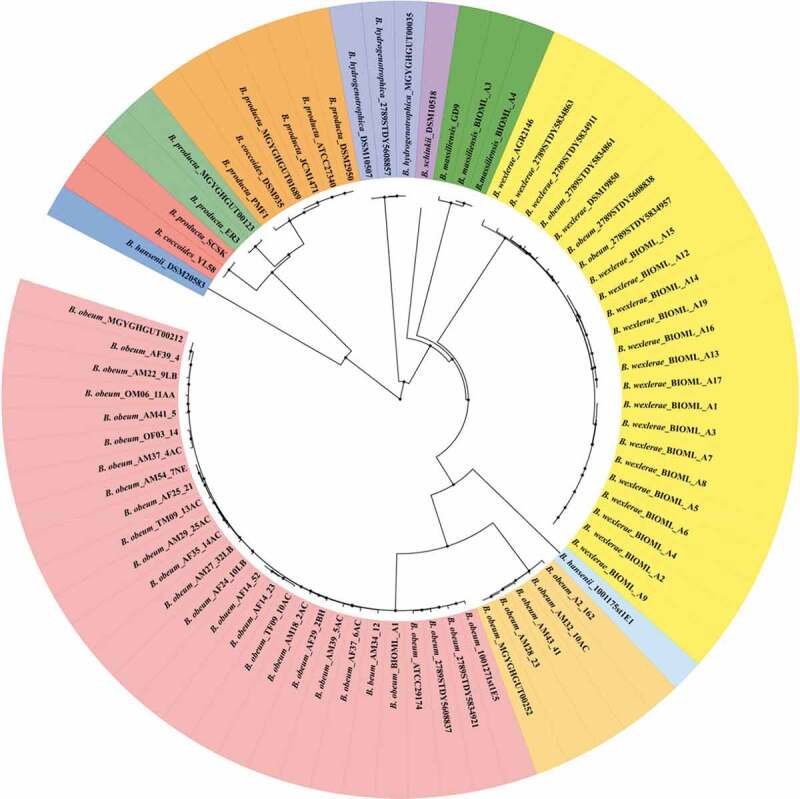
Phylogenetic relationships of different species of *Blautia* based on the orthologous genes. The phylogenetic tree was constructed using the python script3 and visualized using iTOL. Tree nodes are depicted by filled circles. The genomes of 74 *Blautia* strains were obtained from NCBI database

For a better understanding of the differences among different species of *Blautia*, comparative genomic analysis was performed on the 74 strains of *Blautia*, the assembly level of genome sequences of which was more than half (Table S1). Based on the orthologous genes, a phylogenetic tree was constructed to evaluate the evolution of different species (Figure 2). The phylogenetic tree showed that *Blautia* was a paraphyletic group, and all strains were derived from the same ancestor. Different strains within the same species were clustered together except *Blautia hansenii* and *Blautia obeum*.

The Pan-genome is defined as the collection of all genes in a bacterial species, including core genome in all strains and dispensable genome in some strains. The open or closed characteristics of pan-genome could reflect the species diversity in genetic composition.^37^ As shown in Figure 3a, the number of pan-genome increased with the addition of *Blautia* genome, and the total number of pan-genome reached 26,728 when the 74^th^ genome was added to the calculations. The pan-genome curve showed an upward trend, indicating that *Blautia* has an open pan-genome.^38^ In contrast, the core genes curve showed a downward trend, and gradually stabilized at 488 when the 74^th^ genome was added.^39^ The core genes and unique genes of *Blautia* were displayed with a Venn diagram (Figure 3b). The number of core genes was 606, while the number of unique genes ranged from 3 to 995. Notedly, *Blautia schinkii* DSM10518 has the largest number of unique genes, which may be related to the isolation source. Because *B. schinkii* DSM10518 was the only strain isolated from rumen, and the other strains were isolated from human or mouse feces.**Figure 3A.**
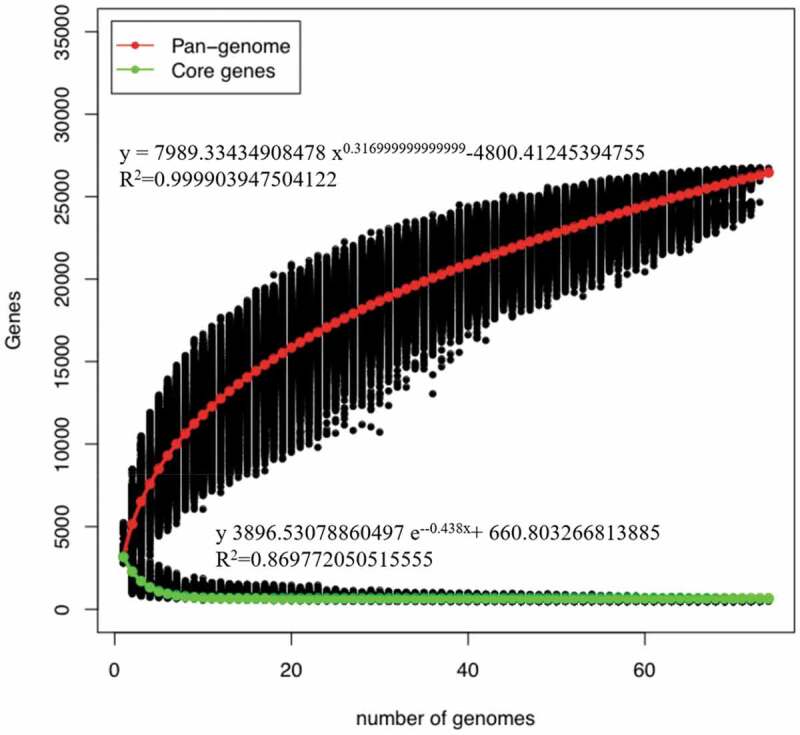
Pan-genome and core genes of *Blautia.*

**Figure 3B.**
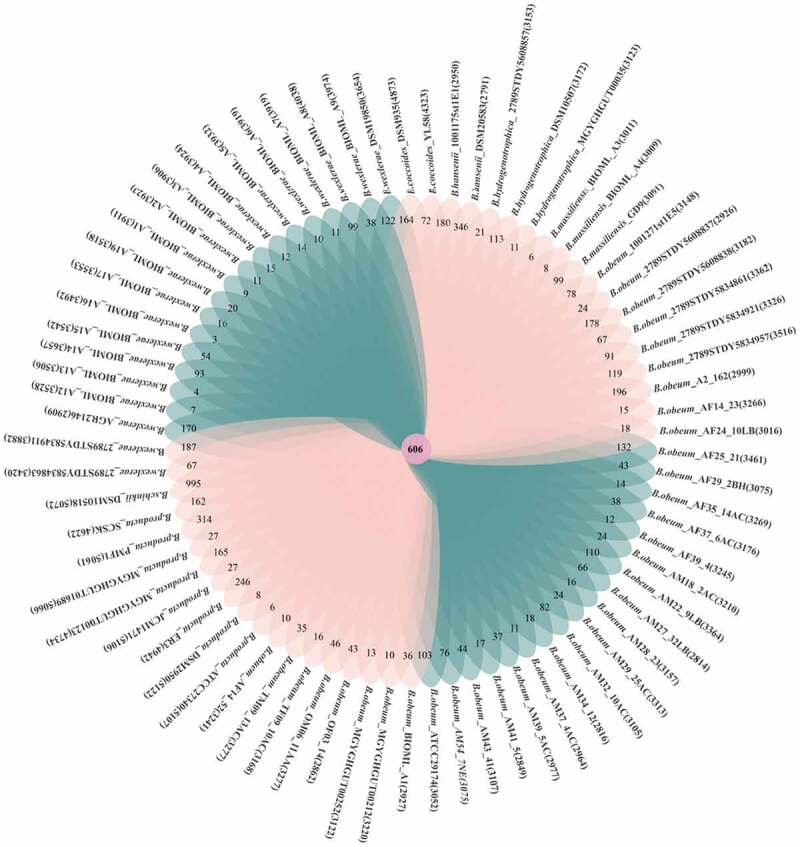
Venn diagram displaying core genes and unique genes of *Blautia* strains

**Figure 3C.**
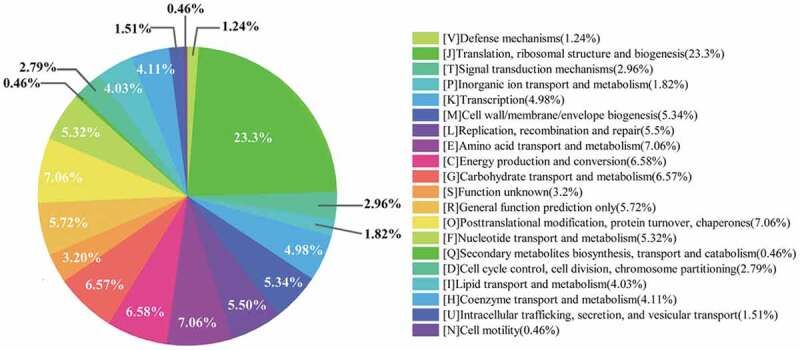
Functional annotations of the core genes using the COG database

**Figure 3D.**
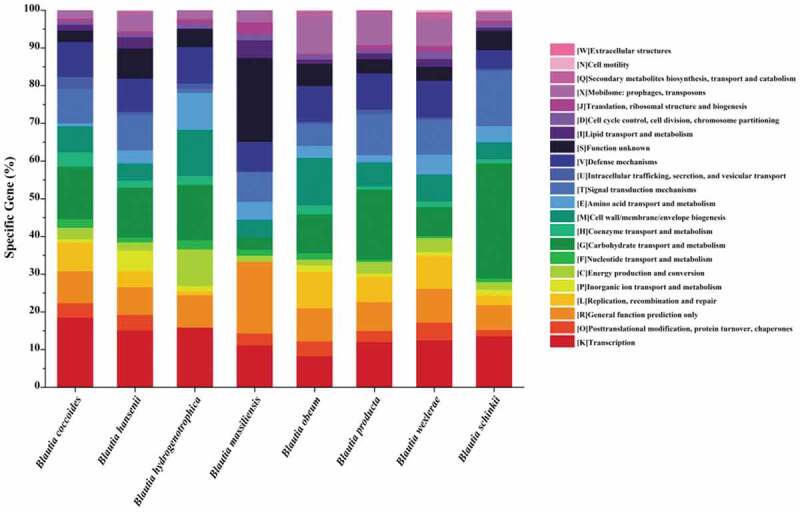
Functional assignment of the unique genes based using the COG database

Based on the clusters of orthologous genes (COG), the core genes of *Blautia* were annotated and evaluated (Figure 3c). The functional genes were roughly divided into 19 categories and were most enriched in [J] Translation, ribosomal structure, and biogenesis (23.30%). The genes for ribosome structure and biogenesis were usually associated with environmental stresses, such as acid, oxidative, heat, and salt stresses.^40–43^ Thus, it may be beneficial for *Blautia* to survive in the harsh conditions in the host gastrointestinal tract due to the large number of stress-related genes.^44^ Compared with most lactic acid bacteria, the proportion of genes related to [G] Carbohydrate transport and metabolism was relatively small in *Blautia* (6.57%), indicating that *Blautia* may have weak abilities to metabolize carbohydrates.

Compared with other species, *Blautia obeum, Blautia producta*, and *Blautia wexlerae* possess more unique genes encoding prophages and transposons (Figure 3d). Prophages allowed bacteria to acquire the antibiotic resistance and enhance the adaption to environment and adhesion ability, which could help the bacteria to cope with multiple adverse environments and colonize in mammals.^45,46^ Remarkably, *Blautia schinkii* isolated from the rumen showed prominent carbohydrate metabolism ability. It is well known that rumen microbiota present in ruminants showed effective utilization of carbohydrates such as fiber and starch in feed, and the special ecological niche would endow strains with unique functions.^47^

## Effects of diet, age, and geography on the abundance of *Blautia*

Diet is the main factor that drives the composition and metabolic activities of intestinal microbiota, as different types and quantities of diet and the balance between major nutrients have a significant effect on the intestinal microbes.^48^ The traditional Japanese cooking method Washoku, which contains fermented food prepared with the nonpathogenic fungus koji, is thought to be closely related to Japanese longevity. One study reported that koji contains a large number of glycosylceramides and showed that the addition of 1% purified glycosylceramides as a prebiotic in the diet of mice for 1 week could improve the abundance of *B. coccoides* in the intestinal tract of mice, reduce their blood sugar level, and upregulate their renal glandular hormone level. *B. coccoides* was found to degrade glycosylceramides to ceramides and then metabolize the ceramides to fatty acids and sphingoid bases, which were absorbed by the intestine and produced a beneficial effect.^49^ The addition of dietary fiber extracted from corn (F-FOPs) to the diet of mice fed a high-fat (HF) diet significantly increased the abundance of *Blautia* in the mouse feces. Compared with mice on the HF diet, those on the F-FOPs + HF diet showed losses of body and tissue weight, and the results revealed a negative correlation between the abundance of *Blautia* and the markers of obesity-related metabolic disorders.^50^ The enrichment of *Blautia* has also been observed in the feces of healthy adult dogs whose diet was supplemented with potato fiber.^51^ Similarly, addition of 20% freeze-dried soy milk to rats fed an HF diet led to an increase in the abundance of *Blautia* in the rat feces. The soy milk was found to regulate the serum high-density lipoprotein-cholesterol level in the rats and enhance the gene expression of tight junction proteins (ZO-1 and occlusion protein) and inflammation-related proteins (IL-1β, IL-10, and Foxp3) in their colon.^52^ Dietary protein and prebiotics from various sources also affect the intestinal microbiota. In one study, 30 female rats were divided into six groups and fed casein or soy protein isolate each with cellulose, raffinose, or fructooligosaccharide (FOS). The results showed that dietary protein from both sources could change the acetic acid concentration and the abundance of *Lactobacillus* in the rat feces, but FOS increased the abundance of *Blautia* regardless of the dietary protein source.^53^ Consistently, another study reported a significant increase in the abundance of *Blautia* in the feces of mice fed FOS.^7^ An increase in the cecal abundance of *B. productus* (previously as *Ruminococcus productus*) was observed in rats fed 3% difructose anhydride III for 2 weeks, accompanied by a decrease in the cecal pH and an increase in the content of short-chain fatty acids (SCFAs). The study also showed that intestinal acidification in rats may inhibit the formation of secondary bile acids.^54^ Furthermore, another study in which a 45-year-old male volunteer consumed 600 mg of omega-3 daily for 14 days, the volunteer’s overall intestinal microbial diversity decreased, accompanied particularly with reduced *Faecalibacterium* abundance and significantly increased *Blautia* abundance.^55^ In addition to diet, the way people eat also shapes the gut microbiota. Compared with the ordinary diet, the alternating diet and the self-service diet can enhance the abundances of *Blautia* and *Ruminococcus* in the gut microbiota, in addition to inducing changes in some host metabolism-related parameters.^56^ With the development of whole-genome sequencing, future studies can examine how various diets regulate the metabolic activities of *Blautia* and improve host health.

Significant changes occur in the gut microbiota during transitions between various stages of life (i.e., from childhood to adulthood to old age). A study that monitored the intestinal microbes of children from 2 weeks to 13 years of age found that *B. coccoides* was rarely present in the intestinal microbiota of children younger 6 months, but was more common in those older than 12 months.^57^ A similar phenomenon was also observed in commercial diet-fed pigs and rats. The researchers found that the abundance of fecal microorganisms continued to change with the age of pigs, accompanied by an increase in the proportion of *Blautia*.^58^ In one study, middle-aged rats and young rats fed the same egg protein diet for 14 days showed significantly different intestinal microbiome at the end of 14 days. Particularly, the middle-aged rats’ relative abundance of *Blautia* was increased.^59^ Another study that analyzed the cecal microbiome of specific pathogen-free chicks on days 14, 28, and 42 by sequencing of the V3-V4 region of 16S rRNA gene on the Illumina MiSeq platform revealed that the abundance of *Blautia* increased with the age of chicks.^60^ A cross-sectional study of the stool samples of 367 healthy Japanese subjects aged 0–104 years using high-throughput sequencing of V3-V4 region of 16S rRNA gene reported that the intestinal microbiota of Japanese adults (21–69 years old) contained high abundance of *Blautia* and *Bifidobacterium* and low abundance of *Bacteroides*.^5^ In addition, compared with the adults, the elderly showed reduced microbiome diversity and abundance of individual microbes, including a lower *Blautia* abundance. This phenomenon may be related to an age-related decline in immune function known as immunosenescence, accompanied by many age-related conditions that involve chronic low-level inflammation.^61^

A recent study analyzed the microbial community characteristics in the stool samples of 303 school-age children from urban or rural areas of five countries in temperate and tropical regions of Asia. The intestinal microbiota of the children was divided into two groups, *Prevotella* (P type) and *Bifidobacterium*/*Bacteroides* (BB type). The gut microbiota of children in China, Japan, Taiwan, and other temperate regions was mostly BB type, whereas that of children in Thailand, Indonesia, and other tropical places was mostly P type. Notably, *Blauti*a was significantly enriched in the BB-type intestinal microbiota, accounting for 10% of the total BB-type bacterial composition but only 5% of the total *P*-type bacterial composition.^6^ Schnorr et al.^62^ found a difference in the intestinal microbial composition between Hadza and Italians, characterized by a lower *Blautia* abundance in Hadza. Differences in the human gut microbiota were also noted between different altitudes and geographies. Sequencing of the fecal microbiota of 208 Tibetans from six regions based on the analysis of the operational taxonomic units revealed *Blautia* to be the dominant genus in the human gut microbiota across all six regions. Further principal component analysis showed that the intestinal microbiota of Tibetans changed significantly with the increase in altitude, body mass index, and age; specifically, the abundance of facultative anaerobes increased. These findings suggest that the intestinal microbiota play an important role in regulating altitude and geographical adaptability.^63^ One study pointed out that the predominant intestinal genera in Japanese people were *Bifidobacterium* and *Clostridium*; that in the American, Chinese, French, and Spanish people was *Bacteroides*; and that in Australians was *Blautia*.^64^ Reportedly, differences in human gut microbial diversity between geographical locations are largely related to heredity, lifestyle, and diet.^65^ Interestingly, *Blautia* was reported of having strong taxonomic association in twin inheritance.^66^ To identify the differences in intestinal microbial communities between human and animal hosts, a study collected fecal samples from seven hosts, including human, pig, cattle, deer, dog, cat, and chicken, and sequenced the V6 region of the 16S rRNA genes. Two hundred high-resolution taxonomic units in *Blautia* were identified using oligotyping, and the *Blautia* oligotypes could accurately identify different host sources, suggesting that the genus has host specificity and host preference.^67^

## Physiological functions of *Blautia*

### Biotransformation of bioactive substances by Blautia

Human intestinal bacteria belonging to the genera *Prevotella* and *Xylanibacter* can degrade dietary components such as cellulose and xylan that are not digested by the host, increase the content of SCFAs in feces, promote food digestion, and maximize energy intake.^68^ In recent years, research on the biotransformation and metabolism of herbal plants and functional foods by *Blautia* has attracted research attention.

Polymethoxyﬂavones (PMFs) are flavonoids isolated from *Kaempferia* and citrus fruits and have anticancer, anti-inflammatory, antiviral, and anticoagulant properties, and other biological functions.^69–72^ Studies have shown that the strain *Blautia* sp. MRG-PMF1 has a hydrolytic effect on aryl methyl ether functional groups by converting 5,7-dimethoxyflavone (5,7-DMF) and 5,7,4-trimethoxyflavone (5,7,4-TMF) into bioactive chrysin and apigenin, respectively. This strain also possessed deglycosylation ability, whereby it can metabolize isoflavones, flavones, and flavonoids into corresponding aglycones.^8^ Wu et al.^73^ found that *Blautia* sp. MRG-PMF1 can also biotransform icariin under anaerobic conditions and metabolize it to its hydrolyzates icariin and desmethylicaritin, which were reported to exhibit estrogenic effects by acting on estrogen receptors, in addition to significant antilipogenic activity. Another study revealed that this strain can also metabolize curcumin to demethylcurcumin and bisdescurcumin.^74^ Compared with curcumin, demethylcurcumin has been found to be highly toxic to human HCT116 colon cancer cells, whereas synthetic desmethylcurcumin has been found to exert better neuroprotective and anti-inflammatory effects.^75–77^ Furthermore, the strain *Blautia* sp. AUH-JLD56 has been shown to specifically and efficiently biotransform arctiin or arctigenin to (−)-3′-desmethyl arctigenin, which possesses good free radical-scavenging activity.^78^ *B. glucerasei* sp. nov. HFTH-1 T produces a specific extracellular glucosylceramide enzyme that hydrolyzes glucosylceramide into functional substances with specific preventive effect against colon cancer (Table 4).^18^ It should be pointed out that some biotransformation by *Blautia* may not be beneficial and could potentially even be harmful. Certain *Blautia* species could perform 7-α-dehydroxylation of primary bile acids and convert them to secondary bile acids such as lithocholic acid and deoxycholic acid, which have been reported as colon cancer-inducing carcinogens and are found in higher concentrations in the feces of patients with ulcerative colitis and dysplasia or cancer.^79^

**Table 4.** Biotransformation of bioactive substances by *Blautia.*

*Blautia* is also involved in the process of polyphenol deglycosylation and lignan catabolism. In general, bacterial metabolism in the intestine does not involve oxygen but rather reductions and hydrolysis, resulting in the formation of nonpolar low molecular weight products. In the course of flavonoid conversion, the reactions catalyzed by *Blautia* include demethylation, dehydroxylation, O-and C- deglycosylation and c-ring cleavage,^80^ which may be due to its corresponding enzymes, such as β-glucosidases and O-glycosidase.^81,82^ By analyzing the key metabolic pathways and enzymes related to biotransformation, it is possible to predict whether the bacteria can undergo the biotransformation of specific bioactive substances. Thus, the exploration of biotransformation by *Blautia* is essential for the development of new enzymes and bioactive metabolites for supplementation in food and provide valuable perspective for the metabonomics research of human intestinal microbiome.^83,84^

### Relationships between Blautia and host health

Intestinal microbiota is a complex ecosystem that is linked to the development of host disease, drug metabolism, immune system regulation, and other processes.^85^ *Blautia*, as a dominant genus in the intestinal microbiota, has a significant correlation with host physiological dysfunctions, such as obesity, diabetes, cancer, and various inflammatory diseases.

#### Blautia and the secondary metabolites

Secondary metabolites are biologically active compounds produced by microorganisms during growth and metabolism and widely used in antibacterial and anticancer drugs, herbicides, and insecticides, which were also an important source of microbial drug development.^86,87^ According to the categories, there were more than 20 kinds of secondary metabolites, such as polyketides (PKS), non-ribosomal peptides (NRPS), lantipeptides/lantibiotics, bacteriocins, and terpenes.^88^ As early as 1980, bacteriocins produced by bifidobacteria were reported to possess antibacterial activity against pathogenic microorganisms such as *Listeria monocytogenes, Clostridium perfringens*, and *Escherichia coli*.^89^ Nisin, which is produced by *Lactococcus lactis*, is used as a natural food preservative.^90^ According to the chemical structures and mechanisms of action, bacteriocins are divided into four classes, and sactipeptides and lanthipeptides are post-translationally modified antibacterial peptides belonging to class I bacteriocins.^91^

**Figure 4.**
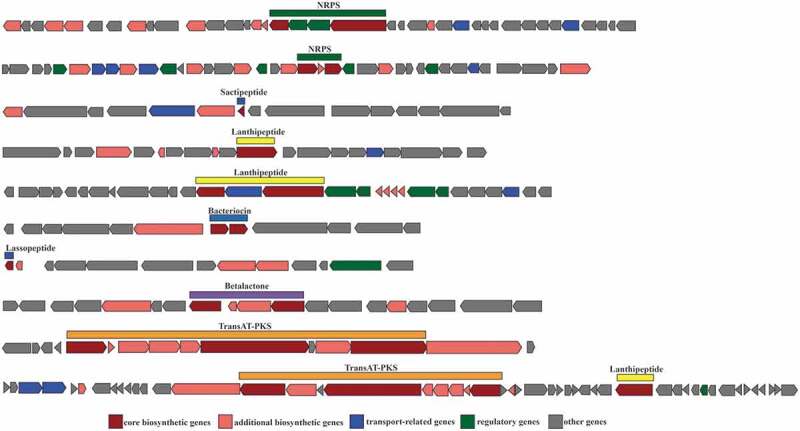
Schematic representation of 10 secondary metabolic biosynthetic gene clusters (BGCs) extracted from 74 *Blautia* genomes

*Blautia* usually has the ability to produce bactericins. Through the annotation of secondary metabolites using the antismash database, 74 strains of *Blautia* were annotated into 7 categories and a total of 261 secondary metabolic biosynthesis gene clusters (BGCs) including NRPS, sactipeptide, lanthipeptide, bacteriocin, lassopeptide, betalactone and transat-pks (Figure 4). NRPs, sactipeptide, lanthipeptide were generally distributed in all the strains (Table S2). NRPs and PKs were among the most profuse families of secondary metabolites with diverse functions, including siderophores involved in iron scavenging, pigments that provide protection against an array of stress factors, as well as nutrient acquisition, chemical communication, and defense responses.^92,93^ Azevedo et al. found that both *Blautia schinkii* DSM 10518 and *Blautia* sp. SF-50 possess a gene cluster that encodes sactipeptide, the structural peptide sequence of which belongs to the protein family TIGR04065. *Blautia* sp. SF-50 also possesses a gene cluster that encodes lanthipeptide.^94^ Hatziioanou et al. found that *B. obeum* A2-162 possess a new antibiotic – nisin O, which contains four structural peptides and an unusual leader peptide sequence and showed antibacterial activity against *C. perfringens* in the presence of trypsin.^95^ In one study, commensal bacteria CBBP_SCSK_ comprising *C. bolteae, B. producta, Bacteroides sartorii*, and *Parabacteroides distasonis* were isolated from mouse feces and were shown to demonstrate colonization resistance to vancomycin-resistant enterococci (VRE).^96^ When the four strains were co-cultured with VRE, only *B. producta* was found to inhibit the growth of VRE by secreting a lantibiotic to exert a bacteriostatic effect. Notably, lanthipeptide has a narrow antibacterial spectrum and specifically targeted VRE without affecting other commensal bacteria. Thus, *B. producta* may serve as a potential probiotic to prevent the infection and transmission of antibiotic-resistant conditional pathogens.^97^

Based on the above reports and analysis of secondary metabolites, we believe that the ability to produce bacteriocins gives *Blautia* the potential to inhibit the colonization of pathogenic bacteria in the intestine, and it can also affect the composition of intestinal microbiota. In particular, *B. obeum* and *B. producta* can inhibit the proliferation of *C. perfringens* and vancomycin-resistant enterococci, which makes it possible to become potential probiotics and exert probiotic functions.

#### Blautia and the obesity-related diseases

In recent decades, the prevalence of obesity-related metabolic syndromes such as type 2 diabetes has dramatically increased worldwide, which has, in turn, increased the risk of atherosclerosis, nonalcoholic fatty liver disease, certain cancers, and other diseases. Recent studies have suggested that the intestinal microbiota play an important role in obesity and related diseases.^98^ In a population-based cross-sectional study, the researchers investigated the relationship of visceral fat accumulation and body mass index in Japanese men and women aged 20–76 years with the intestinal microbiota stratified by sex. *Blautia* was found to be the only genus whose abundance showed a significant negative relationship with visceral fat accumulation in Japanese people, regardless of sex.^99^ Consistently, one study observed a change in the composition of intestinal microbiota and an increase in the abundance of *Blautia* in overweight/obese patients with nonalcoholic fatty liver who consumed a low-calorie and high-protein diet for 3 weeks.^100^ In another study, obese children, regardless of the presence of nonalcoholic steatohepatitis, showed a higher intestinal abundance of *Bacteroide*s and a lower abundance of *Firmicutes*, accompanied by reduced abundances of *Blautia* and *Faecalibacterium*.^101^ A study evaluating the effect of *Panax ginseng* on the weight loss of middle-aged obese women in Korea showed that *Blautia* was a dominant genus in the intestinal microbiota of women in the effective weight loss group but not in those in the ineffective weight loss group.^102^ A previous study evaluating the effects of berberine and metformin on the intestinal microbiota of rats with HF diet-induced obesity demonstrated that berberine and metformin altered the overall structure of the intestinal microbiota; more specifically, a reduction in the diversity of intestinal microbiota but a significant increase in the abundance of SCFAs-producing *Blautia* were observed.^103^ In another study, the abundance of *Blautia* was decreased significantly in children with diabetes compared with healthy children.^104^ Inoue et al.^105^ revealed that *Blautia* abundance was significantly reduced in Japanese patients with type 2 diabetes compared with the control group, indicating a negative correlation between *Blautia* abundance and the levels of fasting plasma glucose and hemoglobin A1C (HbA1c). A previous study showed that the insulin signaling pathway and glycosylation/glycogen metagenesis were upregulated in diabetic patients, and this upregulation was positively correlated with the HbA1c level. Metformin and a specially designed herbal formula (AMC) were used to treat patients with type 2 diabetes for 12 weeks and significant decreases in hyperglycemia and hyperlipidemia were observed, in addition to changes in the structure of intestinal microbiota; specifically, the abundance of *Blautia* was increased, which was associated with the improvements in glucose and lipid homeostasis.^106^ *Blautia* is a common acetic acid producer in the intestine, which may inhibit insulin signaling and fat accumulation in adipocytes by activating the G protein-coupled receptors GPR41 and GPR43, in turn promoting the metabolism of unbound lipids and glucose in other tissues and thus alleviating obesity-related diseases.^107^ A cross-sectional study showed that *Blautia*, especially *B. luti* and *B. wexlerae*, probably help to reduce the inflammation associated with obesity-related complications. Therefore, these new findings will provide valuable information for the microbiota-based strategies for early prevention of obesity-related complications in the future.^108^

#### Blautia and inflammatory diseases

Vibrio cholerae usually causes human diarrhea, but people have different susceptibility to the pathogen, which may be driven by the interpersonal microbiome variation. Alavi et al. found that the intestinal microbiota of cholera patients were significantly different from that of healthy individuals, in which *Blautia obeum* showed a significant negative correlation with the colonization of *Vibrio cholerae*. Further studies showed that the genes encoding bile salt hydrolases (BSH) in *B. obeum* genome could reduce the expression of *tcpA* gene in *V. cholera*, inhibit its colonization and alleviate diarrhea.^109^ Allogeneic blood/bone marrow transplantation (allo-BMT) is a vital therapy for patients with leukemia, lymphoma, and other cancer-related hematological malignancies, but its biggest drawback is that the donor’s immune system recognizes the recipient’s organs as foreign, leading to graft-versus-host disease (GVHD).^110^ A study with biomarker analysis revealed that increased diversity of intestinal microbiota in patients undergoing allo-BMT, particularly increased abundance of symbiotic bacteria of the *Blautia* genus, is associated with a reduction in lethal GVHD and an increase in overall survival.^111^ A few studies have also reported the relationship of decreased abundance of *Blautia* with ileal pouch–anal anastomosis and liver cirrhosis.^112,113^ *Blautia*, as a genus of commensal obligate anaerobic bacteria, plays an important role in maintaining environmental balance in the intestine and preventing inflammation by upregulating intestinal regulatory T cells and producing SCFAs.^114^ Inflammatory bowel disease (IBD), including Crohn’s disease (CD) and ulcerative colitis, is a nonspecific chronic intestinal inflammatory disease of unknown etiology, whose incidence has been increasing in many developing countries in recent decades.^115^ Analysis of fecal and mucosal microbial communities in IBD patients and healthy people has revealed that *Blautia* abundance is significantly reduced in the cecal mucosal microbiota of patients with CD.^116^ A similar decrease in *Blautia* abundance has also been reported in the mucosal adherent microbiota of colorectal cancer patients.^117^ In addition, the abundances of *Faecalibacterium prausnitzii* and *Blautia* have been reported to be reduced in the gut microbiota of patients with colitis-related cancer compared with that in healthy individuals. The study showed that *Blautia* and *F. prausnitzii* accounted for a lower proportion in the intestinal mucosa-associated microbiota of sporadic cancer patients than in that of healthy individuals, but were abundant in the extratumor microenvironment.^118^ *F. prausnitzii* has been shown to exert anti-inflammatory effects on colitis by blocking NF-*k*B expression and IL-8 secretion, as well as inducing colonization resistance against pathogens; these findings suggest that *F. prausnitzii* and *Blautia* exert protective effects against carcinogenesis.^119,120^

The above research shows that the abundance of *Blautia* is negatively correlated with some diseases. However, higher abundance of *Blautia* was found in the fecal microbiota of irritable bowel syndrome and ulcerative colitis patients compared with healthy individuals.^121,122^ A greater abundance of *Blautia* has also been reported in early breast cancer patients with increasing clinical stage and tissue pathological classification (Table 5).^123^ These conflicting conclusions may raise the question that whether *Blautia* is good for human health. In fact, most of these reports focused on the genus level, and did not conduct in-depth studies at the species or even strain levels. We must avoid drawing general conclusions at the genus level. There may be differences in the composition of *Blautia* at the species level, and different species of *Blautia* may exert beneficial or adverse effects on human health.

**Table 5.** Reported roles of *Blautia* in host health

Species/strain	Functions	Significant findings	Reference (author,year)
*B. schinkii* DSM 10518	Antibacterial (bacteriocins)	*B. schinkii* DSM 10518 had a gene cluster encoding Sactipeptide	Azevedo et al., 2015^94^
*Blautia* sp. Strain SF-50		*Blautia* sp. Strain SF-50 had the genes cluster encoding both Sactipeptide and lanthipeptide	
*B. obeum* A2-162	Antibacterial (bacteriocins)	A new lantibiotic nisin O isolated from *B. obeum* A2-162 showed antibacterial activity against Clostridium perfringens	Hatziioanou et al., 2017^95^
*B. producta*	Antibacterial (bacteriocins)	Commensal bacterias CBBP_SCSK_ containing *B. producta* demonstrated colonization resistance to vancomycin-resistant enterococci (VRE)	Caballero et al., 2017^96^
*B. producta*	Antibacterial (bacteriocins)	*B. producta* inhibited the growth of VRE by secreting a lantibiotic similar to nisin-A to exerts bacteriostatic effect	Kim et al., 2019^97^
*Blautia*	Visceral fat accumulation (VFA)	Regardless of gender, *Blautia* was the only genus which significantly negatively related to VFA	Ozato et al., 2019^99^
*Blautia*	obese	For overweight/obese patients with nonalcoholic fatty liver, the intestinal microbiota of the patients changed and the abundance of *Blautia* increased	Pataky et al., 2016^100^
*Blautia*	obese	Obese children showed higher levels of *Bacteroides* and lower levels of *Firmicutes*, accompanied by reduced the number of *Blautia* and *Faecalibacterium*	Zhu et al., 2013^101^
*Blautia*	obese	A study evaluating the effect of Panax ginseng showed that *Blautia* was dominant in the intestinal microbiota of the effective weight loss group than the ineffective weight loss group	Song et al., 2014^102^
*Blautia*	obese	Berberine and metformin alter the overall structure of the intestinal microbiota of obese rats, increasing the abundance of SCFAs – producing bacteria *Blautia*	Zhang et al., 2015^103^
*B. luti and B. wexlerae*	obese	In obesity, the decrease of *B.luti* and *B.wexlerae* species in intestinal ecosystem may lead to metabolic inflammation and insulin resistance	Benitez-Paez et al, 2020^108^
*Blautia*	diabetes	Compard to the healthy children, the abundance of *Blautia* significantly decreased in children with diabetes	Murri et al., 2013^104^
*Blautia*	diabetes	Compard to the control group, *Blautia* was significantly reduced in patients with type 2 diabetes	Inoue et al., 2017^105^
*Blautia* spp	diabetes	Metformin and specially designed herbal formula (AMC) could change the structure of intestinal microbiota and increase the abundance of *Blautia*	Tong et al., 2018^106^
*Blautia*	graft-versus-host disease (GVHD)	In allogeneic blood/bone marrow transplantation (allo BMT) patients, the abundance of *Blautia* was associated with a reduction in lethal GVHD and an increase in overall surviva	Jenq et al., 2015^111^
*Blautia*	ileal pouch-anal anastomosis	The abundance of *Blautia* decreased in ileal pouch anal anastomosi	Tyler et al., 2013^112^
*Blautia*	liver cirrhosis	The abundance of *Blautia* decreased in liver cirrhosis	Kakiyama et al., 2013^113^
*Blautia*	Inflammatory Bowel Disease(IBD)	Compared to the healthy people, the cecal mucosal microbiota *Blautia* was significantly reduced in patients with Crohn’s disease (CD)	Chen et al., 2014^116^
*Blautia*	colorectal cancer (CRC)	There was a decrease in *Blautia* in the mucosal adherent microbiota of colorectal cancer patients	Chen et al., 2012^117^
*Blautia obeum*	Cholera Infection	*Blautia Obeum* could disable the pathogenic mechanism of Vibrio cholerae, preventing it from colonizing the gut	Alavi etal, 2020^109^

In summary, *Blautia* abundance has a close relationship with various diseases, but literature on the accurate evidence of this relationship is currently limited. Despite some conflicting findings and lack of clarity on the potential mechanism of *Blautia* in various diseases, *Blautia* abundance can still be used as a potential tool for the early diagnosis or treatment of related diseases.

### Cross-feeding of Blautia with other microorganisms

When the metabolites produced by bacteria from dietary components act as substrates to support the growth of other species, it is called cross-feeding.^124^ Cross-feeding is an important interaction between anaerobic bacteria in the intestinal microbiota that can affect their metabolic pathways and contribute to their stability and productivity.^125^

As a genus of anaerobic bacteria, the cross-feeding of *Blautia* with other bacteria also contributes to metabolic regulation to some extent. A study found that by using 0.2% resistant starch as an energy source, *R. bromii* produces formic acid, ethanol, and acetic acid in approximately equal molar ratios on RUM-RS medium.^9^ However, batch co-culture with acetogenic bacteria *B. hydrogenotrophica* on starch resulted in the disappearance of formic acid with an increase in acetic acid levels. RNA sequencing was used to further study the interspecific interactions to detect gene expression in continuous co-cultures of *R. bromii* and *B. hydrogenotrophica*. Transcriptome analysis revealed the upregulation of *B. hydrogenotrophica* genes involved in the Wood–Ljungdahl pathway in addition to a 10-gene cluster responsible for increased branched-chain amino acid fermentation in the co-culture. Cross-feeding between formic acid-producing species and acetic acid-producing species may play an important role in the formation of SCFAs in the colon and contribute to the massive production of acetic acid.^9^ As a key hydrogen-consuming anaerobic microorganism, *B. hydrogenotrophica* has been reported to mediate regulation related to the coexistence of anaerobic respiratory pathways. *B. hydrogenotrophica* consumes H_2_ and CO_2_ via the Wood–Ljungdahl pathway to produce acetic acid – a pathway significantly activated when coexisting with *Bifidobacterium bifidum. Bi. bifidum* serves as a special carbohydrate-fermenting species and produces CO_2_, which is a fixed substrate in the Wood–Ljungdahl pathway. Thus, the changes observed in the Wood–Ljungdahl pathway in *B. hydrogenotrophica* may be the result of cross-feeding by *Bi. bifidum*.^126^

## Conclusions

As a dominant genus of intestinal microbiota, *Blautia* plays certain roles in metabolic diseases, inflammatory diseases, and biotransformation. However, most of the properties of this genus are linked with its potential probiotic functions, and the causal relationship between *Blautia* abundance and diseases is not yet clear. In addition, there is a disparity in association of the genus with human diseases (less *Blautia* in sufferers of diabetes/obesity, less in CRC and CD but more in IBD). We could not overgeneralize the effects of *Blautia* at a genus level and should consider the importance of species – and perhaps strain differences. Whether *Blautia* plays a direct regulatory role in diseases requires further intervention studies and more detailed evidence. Meanwhile, there is no clear evidence to support its application in the clinical setting and food industries, and its safety in humans requires further verification. As strictly anaerobic bacteria, *Blautia* requires harsh cultivation conditions with rigorous operation methods. To date, only a few strains of *Blautia* have been isolated, and no description of the characteristics of this genus appears in the Bergey’s manual. Moreover, it still has not attracted adequate attention from the researchers, and its genomic information remains limited. Moreover, *Blautia* has host specificity and the oligotypes could accurately identify different host sources. Thus, more *Blautia* strains need to be isolated from different hosts to characterize the physiological properties of this genus and sequence their genomes to clarify the connection between *Blautia* and host health for a comprehensive understanding. Given the role of *Blautia* in the metabolic regulation in host, the use of prebiotics, such as some oligosaccharides, as substrates to promote the proliferation of *Blautia* also remains to be explored to elucidate its probiotic functions.

## Supplementary Material

Supplemental Material

Supplemental Material
